# Parkinson’s disease prognostic scores for progression of cognitive decline

**DOI:** 10.1038/s41598-019-54029-w

**Published:** 2019-11-25

**Authors:** Galina Gramotnev, Dmitri K. Gramotnev, Alexandra Gramotnev

**Affiliations:** 1 Research and Data Analysis Centre, GPO Box 1272, Aspley, QLD 4034 Australia; 2 University of Sunshine Coast, Thompson Institute, 12 Innovation Parkway, Birtinya, QLD 4575 Australia

**Keywords:** Statistical methods, Data mining, Parkinson's disease, Prognostic markers, Parkinson's disease

## Abstract

Clinical and biochemical diversity of Parkinson’s disease (PD) presents a major challenge for accurate diagnosis and prediction of its progression. We propose, develop and optimize PD clinical scores as efficient integrated progression biomarkers for prediction of the likely rate of cognitive decline in PD patients. We considered 269 drug-naïve participants from the Parkinson’s Progression Marker Initiative database, diagnosed with idiopathic PD and observed between 4 and 6 years. Nineteen baseline clinical and pathological measures were systematically considered. Relative variable importance and logistic regressions were used to optimize combinations of significant baseline measures as integrated biomarkers. Parkinson’s disease cognitive decline scores were designed as new clinical biomarkers using optimally categorized baseline measures. Specificities and sensitivities of the biomarkers reached ~93% for prediction of severe rate of cognitive decline (with more than 5 points decline in 4 years on the Montreal Cognitive Assessment scale), and up to ~73% for mild-to-moderate decline (between 1 and 5 points decline). The developed biomarkers and clinical scores could resolve the long-standing clinical problem about reliable prediction of PD progression into cognitive deterioration. The outcomes also provide insights into the contributions of individual clinical and pathological measures to PD progression, and will assist with better-targeted treatment regiments, stratification of clinical trial and their evaluation.

## Introduction

Parkinson’s disease (PD) is a clinically and biochemically heterogeneous neurodegenerative disorder whose diagnosis, prognosis and evaluation of the likely progression remain essentially clinical and present significant challenge^1–4^. So far, there are no accepted diagnostic or progression biomarkers for PD^2,5–7^. The difficulties with the development of biomarkers for this disorder are largely related to heterogeneity of PD, its poor clinicopathological correlation and instability of the clinical phenotypes, and significant overlaps of the clinical and biochemical characteristics with healthy controls and patients suffering from other neurodegenerative disorders^1,2,5–9^. This significantly impedes optimal therapy advice and evaluation of new drugs and therapies for PD.

Significant focus of the search for PD biomarkers has been on the identification of suitable individual clinical or biochemical measures (markers). These included biochemical compounds in the cerebrospinal fluid (CSF), including such potential biomarkers as alpha synuclein (α-syn), total tau (t-tau), phosphorylated tau 181 (p-tau), and beta-amyloid 1–42 (Aβ_42_)^3,6,10–15^. The other significant group of measures as potential PD biomarkers were derived from blood, including insulinlike growth factor 1 (IGF-1) and epidermal growth factor (EGF)^1,3,14–18^, and from other peripheral tissue biopsies^3,18^. Genetic markers^19,20^ and dopamine transporter (DaT) imaging^21,22^ were also considered as potential biomarkers for PD diagnosis and progression.

However, despite the apparent success with identifying numerous individual measures that could aid with PD diagnosis and prognosis, it has become apparent that no such measure could be an efficient biomarker for this disorder and its progression^2,6,23^. This is because no individual measure is capable of reflecting the vast heterogeneity of the clinical and biochemical presentation of this disease. For example, although DaT imaging was recently indicated by the European Medicines Agency and Food and Drug Administration as an ‘enrichment biomarker’ for inclusion in clinical trials^24^, significant deficiencies and lack of reliability of this biomarker (when it comes to prediction of PD progression) have also been highlighted^25^. A way out of these difficulties has been seen in simultaneous use of multiple individual measures to ensure more accurate PD diagnosis and prognosis^2,6,23^. It is expected that integrated biomarkers constructed as combinations of individual measures could be capable of capturing and properly reflecting the heterogeneous nature of PD, thus enabling its reliable prognosis. In integrated biomarkers, failure of individual measures as PD markers for a particular patient could be effectively compensated by other better-performing measures.

It was demonstrated that integrated biomarkers constructed as combinations of multiple CSF measures could be efficient in distinguishing PD patients from healthy controls and from patients with multiple system atrophy and Alzheimer disease^26^. Other integrated biomarkers for PD diagnosis combined such measures as olfactory function determined by the University of Pennsylvania Smell Identification Test (UPSIT) score, age, gender, family history of PD and genetic risk score (GRS)^27^, and several different CSF metabolites^28^. Integrated biomarkers were also described for prediction of cognitive impairment and dementia in PD patients within 2 and 10 years of the diagnosis^29,30^. However, the choices of the clinical and pathological measures for the developed integrated biomarkers^26–30^ were not sufficiently justified or optimized. The effects of age and other clinical and pathological measures are still unclear because of their possible non-linearities that were not considered in the previous publications. Finally, the previously proposed integrated PD biomarkers^26–30^ resulted in rather complex evaluating techniques with little chance of their direct clinical application.

Our goal is to use 19 commonly evaluated clinical and pathological measures to systematically construct and optimize integrated biomarkers for prediction of severe and mild-to-moderate rates of cognitive decline among patients with PD. This will include the development and characterization of Parkinson’s Disease Cognitive Decline (PDCD) scores as new clinical progression biomarkers. These scores that are similar to the Framingham Risk Score (assessing the 10-year risk of cardiovascular disease^31,32^) and DRAGON score (predicting outcomes for ischemic stroke patients^33^) will enable simple clinical evaluation of risks for newly diagnosed PD patients to experience severe or mild-to-moderate rates of cognitive decline.

## Study Participants

The data for this study was obtained from the Parkinson’s Progression Marker Initiative (PPMI) sponsored by the Michael J. Fox Foundation for Parkinson’s Research^34^. The PPMI database contained 269 participants who satisfied the following criteria for inclusion in the current study: (1) all of them were diagnosed with idiopathic PD within 2 years prior the initial screening visit and record of their baseline characteristics in the PPMI database^13^; (2) the period of the subsequent observation of each participant was no less than 48 months, (3) none of them were treated for PD prior to recording their baseline characteristics in the database; (4) all of them had evidence of dopamine deficiency (determined by means of DaT imaging^35–37^); and (5) cognitive function of each participant was evaluated at least 4 times during the period of observation. For some participants, certain measures were not available in the PPMI database, and this constituted missing values. The numbers of observations for the considered variables in this study are presented in Supplementary Table 1.

As was explained above in the Introduction, early accurate diagnosis of PD is difficult because of its heterogeneity and poor distinguishability from other DaT deficit Parkinsonian syndromes, including dementia with Lewy bodies, progressive supranuclear palsy, multiple system atrophy, and cortical basal syndrome^35,36^. Therefore, it was acknowledged that the PPMI PD cohort used in the current study is likely to include a small number of participants with the indicated atypical Parkinsonian syndromes, although at each study visit (at PPMI) the PD diagnosis was reassessed to identify and exclude non-PD subjects^35^. This aspect could be regarded in two different ways. Firstly, this could be considered as a limitation of the current study, if only PD patients are of interest. Alternatively, the developed integrated biomarkers could be regarded as predictors of the rate of cognitive decline among patients who has early-stage PD or another mimicking syndrome (which is still of significant clinical importance). More information on the selection of the study participants can be found elsewhere^35^.

## Results and Discussions

### Variables

Global cognitive function of the study participants and its decline over time were evaluated using the Montreal Cognitive Assessment (MoCA) scale, which is often used for the evaluation of global cognitive function in PD patients^29,30^. As indicated in the Introduction, integrated biomarkers were described for prediction of cognitive impairment and dementia in PD patients within 2 and 10 years of the diagnosis^29,30^. However, prediction of cognitive impairment or dementia developing within a certain time interval^29,30^ may not always be a reflection of the severity of PD progression. Patients may reach cognitive impairment but not experience rapid or severe progression (rate) of their cognitive decline, if their baseline (pre-existing) cognitive state was already low. On the other hand, patients who do not reach cognitive impairment within a specified time interval could still be progressing in their cognitive decline rapidly if their baseline cognitive state was high. Such cases may not be reliably captured by the integrated biomarkers for prediction of cognitive impairment^29,30^. Therefore, the dependent variable adopted in this study was the rate of cognitive decline (RoCD), defined for each participant as the negative coefficient of the linear regression fitted to the changing with time MoCA scores. Thus, RoCD was the average reduction of the MoCA score within one month.

The RoCD variable was categorized to reasonably differentiate between severe and mild-to-moderate cognitive declines. As follows from Supplementary Fig. 1, just under 10% of patients with early stages of PD had RoCD > 0.11 month^−1^, which corresponds to the average decline of their MoCA scores by more than 5 points in 4 years. Such rate of cognitive decline was regarded as severe, as it is likely to cause cognitive impairment within around 4 years, even where the baseline MoCA score (MoCA_b_) is high. At the same time, the values of RoCD between 0.02 month^−1^ and 0.11 month^−1^ (corresponding to the 4-year decline in the MoCA score between 1 and 5 points) were regarded as mild-to-moderate cognitive decline. The proportion of participants with mild-to-moderate cognitive decline was about 30% (Supplementary Fig. 1).

Twenty-two predictor variables were considered, including clinical measures, CSF measures, blood plasma measures, and dopamine transporter single-photon emission computer tomography (DaT) measures^37^ including the caudate-to-background (DaT_c_) and putamen-to-background (DaT_p_) specific binding ratios. The clinical measures included age at baseline, gender, years of prior education, past or present depression and/or anxiety (DA) as a medical condition, baseline MoCA score (MoCA_b_), baseline Geriatric Depression Scale (GDS) score, baseline total State-Trait Anxiety Inventory (STAI) score, baseline University of Pennsylvania Smell Identification Test (UPSIT) score, baseline combined score for sections 1, 2 and 3 of the Movement Disorder Society Unified Parkinson’s Disease Rating Scale (UPDRS_1–3_), genetic risk score (GRS) calculated by summing the risk allele counts for the 30 variants (see the Methods section below) associated with risk of PD^27,38^, and baseline rapid eye movement sleep behavior disorder (RBD) score. The CSF measures at baseline included α-syn, t-tau, p-tau, p-tau/t-tau, and Aβ_42_. The collection process for the CSF measures was described elsewhere^13^. Baseline plasma measures included EGF, IGF-1, triglycerides, and cholesterols. The summary description of the 22 predictor variables used in this study, including their corresponding observation numbers, mean and median values, and the respective standard deviations, are presented in Supplementary Table 1.

### Relative variable importance

**Figure 1:**
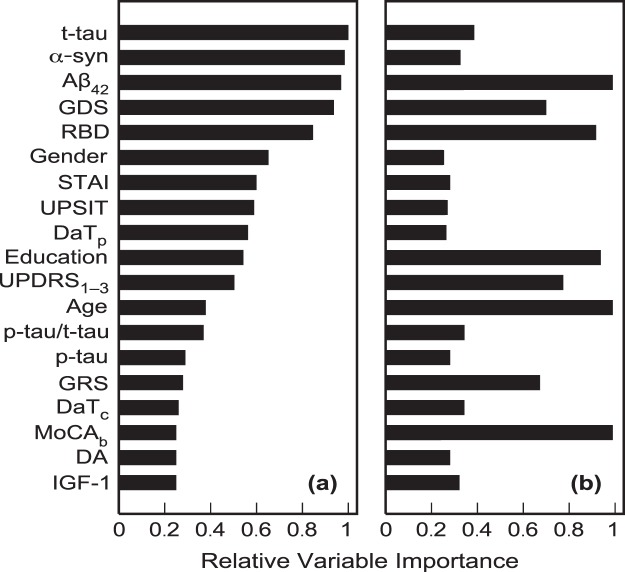
Relative variable importance (probabilities for the variables to appear in the most probable model) for prediction of: (**a**) severe cognitive decline with RoCD > 0.11 month^-1^; and (**b**) decline with RoCD > 0.02 month^−1^.

Relative variable importance for the 19 variables (excluding EGF, triglycerides and cholesterols) obtained using the model averaging approach (see Methods below) is shown in Fig. 1. EGF, triglycerides and cholesterols had limited sample sizes of 123 (Supplementary Table 1), which is why they could not be consistently involved in the model averaging procedure with all other variables. However, the analysis of these three variables within the available limited samples and involving various subsets of other predictors indicated their low importance for prediction of probabilities of the adopted characteristic levels of cognitive decline. Therefore, these three variables were not considered any further in this study.

It can be seen that the sets of important variables for prediction of the severe and mild-to-moderate cognitive declines are significantly different (Fig. 1). The most important variables for predicting the severe cognitive decline (with RoCD > 0.11 month^−1^) are t-tau, α-syn, Aβ_42_, GDS, RBD, Gender, STAI, UPSIT, DaT_p_, years of prior education, and UPDRS_1–3_, and (Fig. 1a). These variables have probabilities of around 50% or greater to appear in the most probable model predicting severe cognitive decline (Fig. 1a). At the same time, the most important variables (with the probability more than 50% to appear in the most probable model) for predicting RoCD > 0.02 month^−1^ include Aβ_42_, Age, MoCA_b_, years of prior education, RBD, UPDRS_1–3_, GDS, and GRS (Fig. 1b). This suggests that integrated markers predicting the severe and mild-to-moderate cognitive declines should be significantly different and must be based on different sets of variables.

### Logistic regressions

The outcomes of the multiple logistic regressions determining significant effects (with *p* < 0.1) of the considered 19 variables on the probabilities for the patients with early stages of PD to experience RoCD > 0.11 month^−1^ and RoCD > 0.02 month^−1^ are shown in Table 1. Any categorization was chosen/optimized to ensure maximum significance of the categorized variables. Any missing coefficients in Table 1 indicate that these coefficients were not significant. The significant variables in Table 1 are not limited to those indicated by their high relative variable importance (Fig. 1). Although the development of the logistic regressions was informed by the outcomes in Fig. 1, all 19 predictors were additionally checked directly for their significance in numerical and categorical forms. The significant intrinsic similarities between the sets of variables that were significant in the developed logistic regressions (Table 1) and those identified by the model averaging approach (Fig. 1) further corroborated the validity of both the methods and their outcomes for the considered data.

**Table 1:** Logistic regression outcomes for the significant effects of the clinical and pathological measures on the probabilities of RoCD > 0.11 month^−1^ and RoCD > 0.02 month^−1^ in patients with early stages of PD.

Predictor variables	RoCD > 0.11 month^−1^		RoCD > 0.02 month^−1^	
	Coefficient	*p*-value	Coefficient	*p*-value
Aβ_42_ (pg/ml)	− 0.0156	0.013	−0.00559	0.005
α-syn (pg/ml)	− 0.00363	0.005		
α-syn < 1950 pg/ml (base: α-syn ≥ 1950 pg/ml)			1.05	0.015
t-tau (pg/ml)	0.150	0.001		
t-tau > 60 pg/ml (base: t-tau ≤ 60 pg/ml)			1.17	0.039
p-tau ≥ 17 pg/ml (base: p-tau < 17 pg/ml)	2.66	0.026		
p-tau / t-tau			−8.89	0.007
(p-tau / t-tau)^2^			7.63	0.007
MoCA_b_ (score at baseline)			0.257	0.002
UPDRS_1–3_ (score at baseline)	0.0633	0.074	0.0302	0.028
RBD (score at baseline)	0.339	0.090	0.161	0.019
GDS (score at baseline)	0.457	0.022	0.132	0.079
STAI (score at baseline)	0.109	0.055		
Gender (base: male)	− 3.64	0.067		
Education (years)			−0.151	0.010
Age (years)			−13.53937	0.014
Age^2^			0.3564239	0.014
Age^3^			– 0.0040239	0.016
Age^4^			0.0000166	0.018
GRS < – 0.01 (base: GRS ≥ – 0.01)			0.758	0.060
DaT_p_ (putamen average)	2.94	0.074		

Samples: 232 participants (RoCD > 0.11 month^−1^); 241 participants (RoCD > 0.02 month^−1^).

**Figure 2:**
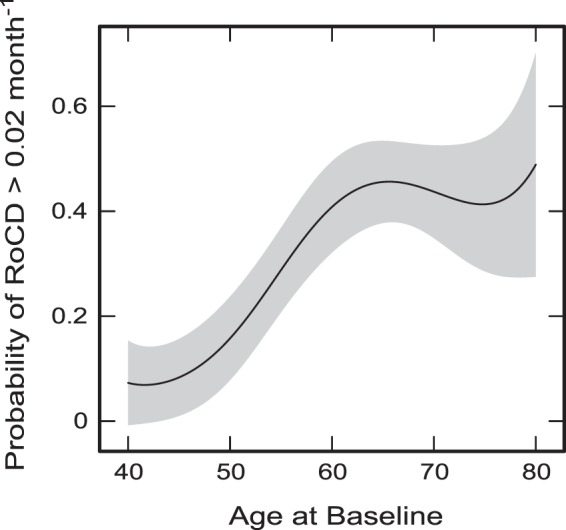
The predicted dependence of the probability of RoCD > 0.02 month^−1^ on baseline age, adjusted to average values of all other significant variables included in the model for RoCD > 0.02 month^−1^ in Table 1. The shaded band shows the 95% prediction interval for the predicted dependence.

Interactions between the variables involved in the models were also checked, including any possible non-linear effects. Significant non-linear effects of Age and p-tau/t-tau were included in the model for prediction of RoCD > 0.02 month^−1^ (Table 1). Age at baseline was previously regarded as the main predictor variable, and its combination with different groups of other baseline clinical measures, CSF parameters, genetic characteristics, and DaT imaging parameters were considered^29,30^. However, the uncovered significant non-linearities of age effects on cognition (Table 1) raise questions about the accuracy of the previously obtained outcomes. The non-linear dependence of the probability/risk of RoCD to exceed 0.02 month^−1^ on baseline age is shown in (Fig. 2), with the largest slope between 48 and 62 years. Above 62 years, there was no significant dependence of the probability of cognitive decline on age (Fig. 2).

It can be seen that the GRS variable is not important for prediction of the severe rate of cognitive decline (Fig. 1 and Table 1), but it is significant (under 10%) for prediction of mild-to-moderate RoCD (Table 1). Because the GRS variable might not be easily available in the clinical practice, we also considered the multiple logistic regression model for RoCD > 0.02 month^−1^ in the absence of the GRS variable. In this case the model presented in Table 1 for RoCD > 0.02 month^−1^ remained essentially the same – with the same characteristic levels of significance of the remaining variables and variations of the regression coefficients under 4%.

### Integrated biomarkers for PD progression

The linear combinations of the significant variables/measures in Table 1 weighed by their respective regression coefficients were considered as the integrated PD progression biomarkers for the severe and mild-to-moderate cognitive declines. For the biomarker predicting RoCD > 0.02 month^−1^, we have:

1 $$\begin{array}{ccc}{M}_{m} & = & -0.00559\times {A\beta }_{42}+1.05\,({\rm{i}}{\rm{f}}\,\alpha {\textstyle \text{-}}{\rm{s}}{\rm{y}}{\rm{n}} < 1950\,{\rm{p}}{\rm{g}}/{\rm{m}}{\rm{l}})\\  &  & +\,1.17({\rm{i}}{\rm{f}}\,{\rm{t}}{\textstyle \text{-}}{\rm{t}}{\rm{a}}{\rm{u}} > 60\,{\rm{p}}{\rm{g}}/{\rm{m}}{\rm{l}})-8.89\times \frac{{\rm{p}}{\textstyle \text{-}}{\rm{t}}{\rm{a}}{\rm{u}}}{{\rm{t}}{\textstyle \text{-}}{\rm{t}}{\rm{a}}{\rm{u}}}+7.63\times {(\frac{{\rm{p}}{\textstyle \text{-}}{\rm{t}}{\rm{a}}{\rm{u}}}{{\rm{t}}{\textstyle \text{-}}{\rm{t}}{\rm{a}}{\rm{u}}})}^{2}\\  &  & +\,0.257\times {{\rm{M}}{\rm{o}}{\rm{C}}{\rm{A}}}_{{\rm{b}}}+0.0302\times {{\rm{U}}{\rm{P}}{\rm{D}}{\rm{R}}{\rm{S}}}_{1{\textstyle \text{-}}3}+0.161\times {\rm{R}}{\rm{B}}{\rm{D}}\\  &  & +0.132\times {\rm{G}}{\rm{D}}{\rm{S}}-0.151\times {\rm{E}}{\rm{d}}{\rm{u}}{\rm{c}}{\rm{a}}{\rm{t}}{\rm{i}}{\rm{o}}{\rm{n}}-13.53937\times {\rm{A}}{\rm{g}}{\rm{e}}\\  &  & +\,0.3564239\times {{\rm{A}}{\rm{g}}{\rm{e}}}^{2}-0.0040239\times {{\rm{A}}{\rm{g}}{\rm{e}}}^{3}+0.0000166\times {{\rm{A}}{\rm{g}}{\rm{e}}}^{4}\\  &  & +\,0.758\times {\rm{G}}{\rm{R}}{\rm{S}}\end{array}$$

whereas for the biomarker predicting RoCD > 0.11 month^−1^ (severe cognitive decline):

2 $$\begin{array}{ccc}{M}_{s} & = & -0.0156\times {{\rm{A}}{\rm{b}}}_{42}-0.00363\times \alpha {\textstyle \text{-}}{\rm{s}}{\rm{y}}{\rm{n}}+0.150\times {\rm{t}}{\textstyle \text{-}}{\rm{t}}{\rm{a}}{\rm{u}}\\  &  & +\,2.66({\rm{i}}{\rm{f}}\,{\rm{p}}{\textstyle \text{-}}{\rm{t}}{\rm{a}}{\rm{u}}\ge 17\,{\rm{p}}{\rm{g}}/{\rm{m}}{\rm{l}})+0.0633\times {{\rm{U}}{\rm{P}}{\rm{D}}{\rm{R}}{\rm{S}}}_{1{\textstyle \text{-}}3}+0.339\times {\rm{R}}{\rm{B}}{\rm{D}}\\  &  & +\,0.457\times {\rm{G}}{\rm{D}}{\rm{S}}+0.109\times {\rm{S}}{\rm{T}}{\rm{A}}{\rm{I}}-3.64({\rm{i}}{\rm{f}}\,{\rm{f}}{\rm{e}}{\rm{m}}{\rm{a}}{\rm{l}}{\rm{e}})\\  &  & +\,2.94\times {{\rm{D}}{\rm{a}}{\rm{T}}}_{{\rm{p}}}.\end{array}$$

**Figure 3:**
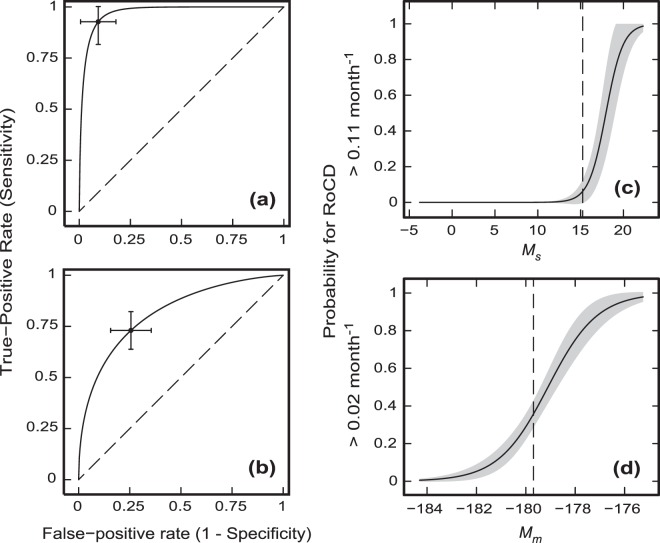
ROC regressions including the marker cut-off points with their respective 95% confidence intervals for the sensitivities and specificities for: (**a**) RoCD > 0.11 month^−1^; and (**b**) RoCD > 0.02 month^−1^. Subplots (**c**,**d**) show the dependences of probabilities of RoCD > 0.11 month^−1^ and RoCD > 0.02 month^−1^, respectively, as functions of the values of the integrated biomarkers *M*_*s*_ and *M*_*m*_ defined by Eqs. (1) and (2). The shaded bands show the 95% prediction intervals for the probability curves, and the vertical dashed lines indicate the positions of the marker cut-off points: *M*_*sc*_ = 15.26 and *M*_*mc*_ = − 178.69.

Figures 3a,b show the outcomes of the quantitative analysis of these biomarkers using the ROC regression analysis, with the corresponding areas under the curve (AUC), sensitivities (Se) and specificities (Sp) presented in Table 2 for the *M*_*s*_ and *M*_*m*_ markers. It can be seen that the marker *M*_*s*_ for prediction of RoCD > 0.11 month^−1^ (severe cognitive decline) has excellent sensitivity and specificity (both above 90% – Table 2). The sensitivity and specificity for the marker *M*_*m*_ predicting RoCD > 0.02 month^−1^ are 73% and 74%, respectively (Table 2).

**Table 2:** Quantitative characteristics (AUC, sensitivity (Se), specificity (Sp), and their respective 95% confidence intervals) for the developed biomarkers *M*_*s*_ and *M*_*m*_ and the respective PDCD scores [with the contributing regression coefficients rounded to the nearest half-integers (index 0.5) and integers (index 1) – see the next section on the PDCD scores].

RoCD	Marker	AUC	95%CI	Se	95%CI	Sp	95%CI
>0.11 month^−1^	*M*_*s*_	0.97	(0.95; 1)	0.93	(0.82; 1)	0.91	(0.82; 1)
	(*PDCD*_*s*_)_0.5_	0.98	(0.96; 1)	0.93	(0.82; 1)	0.93	(0.85; 1)
	(*PDCD*_*s*_)_1_	0.98	(0.96; 1)	0.92	(0.81; 1)	0.94	(0.87; 1)
>0.02 month^−1^	*M*_*m*_	0.81	(0.76; 0.87)	0.73	(0.64; 0.82)	0.74	(0.64; 0.84)
	(*PDCD*_*m*_)_0.5_	0.79 [0.78]	(0.73; 0.85) [0.72; 0.84]	0.73 [0.78]	(0.63; 0.82) [0.69; 0.87]	0.70 [0.63]	(0.61; 0.80) [0.52; 0.73]
	(*PDCD*_*m*_)_1_	0.79 [0.77]	(0.73; 0.85) [0.71; 0.83]	0.67 [0.75]	(0.57; 0.77) [0.66; 0.84]	0.76 [0.65]	(0.67; 0.85) [0.55; 0.76]

AUCs, sensitivities and specificities in square brackets are for the versions of *PDCD*_*m*_ without GRS.

The integrated biomarkers *M*_*s*_ and *M*_*m*_ (Eqs. (1) and (2)) were developed for RoCD > 0.11 month^−1^ and RoCD > 0.02 month^−1^. For each of the two cases, optimized sets of variables and measures constituting the integrated biomarkers *M*_*s*_ and *M*_*m*_ were determined (Eqs. (1) and (2)). In particular, it was shown that the optimized sensitivities and specificities were notably higher for RoCD > 0.11 month^−1^ compared to RoCD > 0.02 month^−1^ (Table 2). As indicated above, the selection of these RoCD categories was reasonable, as these categories contained reasonable proportions of the study participants (Supplementary Fig. 1) and reflected distinctly different progressions of cognitive decline associated with PD.

Supplementary Fig. 2 illustrates variations of the sensitivities and specificities of the optimally constructed integrated biomarkers, if the boundary values of the RoCD categories (for the severe and mild-to-moderate cognitive declines) are different from 0.02 month^−1^ and 0.11 month^−1^. Optimal construction of the biomarkers means that, for each boundary value of RoCD, the corresponding set of significant independent variables was constructed and optimized independently (by maximizing the levels of significance of the independent variables in the model). As a result, it was shown that the sensitivities and specificities of the optimized integrated biomarkers monotonically (albeit non-linearly) increase from their values for *M*_*m*_ for RoCD_0_ = 0.02 month^−1^ to the values for *M*_*s*_ for RoCD_0_ = 0.11 month^−1^ (Supplementary Fig. 2), which further corroborated the consistency of the obtained outcomes.

The integrated biomarkers *M*_*s*_ and *M*_*m*_ should be used in sequence. For example, firstly, we may use the *M*_*m*_ biomarker for a particular PD patient to predict the probability of RoCD > 0.02 month^−1^. If this probability is low, then the patient is unlikely to develop cognitive decline. If this probability is large, the patient is likely to experience mild-to-moderate decline *or* severe cognitive decline. In this case the *M*_*s*_ integrated biomarker (Eq. (2)) should be used to determine the probability for the patient to have severe cognitive decline. If this probability is low, then the conclusion should be that the patient is likely to develop mild-to-moderate cognitive decline (with 0.02 month^−1^ < RoCD ≤ 0.11 month^−1^). If, however, this probability is large, then the final conclusion should be that the patient is likely to develop severe cognitive decline (with RoCD > 0.11 month^−1^). Using this procedure, it will be possible to categorize any PD patient as not likely to have any cognitive decline (RoCD ≤ 0.02 month^−1^), or as likely to have mild-to-moderate cognitive decline (0.02 month^−1^ < RoCD ≤ 0.11 month^−1^), or as likely to have severe cognitive decline (RoCD > 0.11 month^−1^). The probabilities of experiencing RoCD > 0.11 month^−1^ and RoCD > 0.02 month^−1^ as functions of *M*_*s*_ and *M*_*m*_, respectively, are shown in Figs. 3c,d. The precise definition of thresholds for ‘low’ and ‘large’ probabilities is beyond the scope of the current paper and should be based upon a reasonable clinical convention.

Validity of the developed biomarkers *M*_*s*_ and *M*_*m*_ (Eqs. (1) and (2)) was further confirmed by way of their cross-validation using the available cohort of participants (see the Supplementary Information and Supplementary Fig. 3). Control for blood contamination of the CSF samples by removing participants whose baseline CSF samples contained hemoglobin levels exceeding 200 ng/ml^13^ did not cause any significant alterations in the developed models. This demonstrated good stability of the conducted analyses, and blood contamination in the considered database was not a significant issue for the development of the integrated biomarkers (Eqs. (1) and (2)). However, it is important to note that application of these biomarkers to patients with significant blood contamination of their CSF samples could cause significant prognostic errors. Therefore, clinical use of the developed biomarkers requires CSF samples free from significant blood contamination (with hemoglobin levels below 200 ng/ml^13^).

### Parkinson’s disease cognitive decline (PDCD) scores

The complexity of the integrated biomarkers *M*_*s*_ and *M*_*m*_ (Eqs. (1) and (2)) could be a hindrance for their effective clinical use. The significant success and clinical impacts of the previously developed Framingham Risk Score for prediction of risk of coronary heart disease^31,32^ and DRAGON score for prognosis of acute ischemic stroke patients^33^ motivated the development and validation of similar simplified clinical scores for Parkinson’s disease cognitive decline (PDCD).

To derive the PDCD scores, all numerical variables in the logistic regressions (Table 1) were categorized to obtain characteristic ranges (categories) for these variables. The boundaries for the variable categories (Table 3) were determined and optimized to ensure maximum significance of the categorized variables in the respective logistic regression models. Numerical variables that were not significant in Table 1 were also categorized and their significance was further checked in the models with the categorized variables. The outcomes of the logistic regressions with the categorized variables are presented in Table 3. Compared to Table 1 for the numerical biomarkers, Table 3 does not contain Gender and DaT_p_. This is because these variables were not significant in the model with the categorized variables.

**Table 3:** Logistic regression outcomes for significant effects of the categorized clinical and pathological parameters/measures on probabilities of RoCD > 0.11 month^−1^ and RoCD > 0.02 month^−1^ in patients with early stages of PD.

Predictor	RoCD > 0.11 month^−1^ Categories	RoCD > 0.11 month^−1^		RoCD > 0.02 month^−1^ Categories	RoCD > 0.02 month^−1^	
		Coef	*p*-value		Coef	*p*-value
Aβ_42_ (pg/ml)	(1) < 255 (base: ≥255)	2.54	0.020	(1) < 420 (base: ≥420)	0.858 [0.793]	0.040 [0.049]
α-syn (pg/ml)	(1) < 1060 (base: ≥1060)	8.15	0.004	(1) < 1950 (base: ≥1950)	1.07 [1.11]	0.009 [0.006]
t-tau (pg/ml)	(1) > 75 (base: ≤75)	5.74	0.007	(1) > 60 (base: ≤60)	1.37 [1.38]	0.011 [0.010]
p-tau (pg/ml)	(1) ≥ 17 (base: < 17)	4.73	0.008	—	—	—
p-tau/t-tau	(1) < 0.55 (base: ≥ 0.55)	7.10	0.034	(1) < 0.3 (base: ≥0.3)	0.64 [0.63]	0.045 [0.047]
UPDRS_1–3_ (at baseline)	(1) > 49 (base: ≤49)	3.77	0.020	(1) > 43 (base: ≤43)	0.92 [0.92]	0.031 [0.027]
GDS (at baseline)	(1) > 3 (base: ≤3)	2.93	0.023	(1) > 1 (base: ≤ 1)	0.66 [0.58]	0.043 [0.70]
Education (years)	(1) ≤ 10 (base: > 10)	4.20	0.060	(1) ≤ 12 (base: > 12)	1.04 [0.92]	0.014 [0.026]
GRS (at baseline)	—	—	—	(1) < – 0.01 (base: ≥– 0.01)	0.97 [−]	0.017 [−]
MoCA_b_ (at baseline)	—	—	—	(1) > 27 (base: ≤27)	0.94 [0.92]	0.006 [0.006]
Age (years)	—	—	—	(1) 52 < Age ≤ 62; (2) Age > 62 (base: ≤52)	(1) 1.32 [1.32] (2) 2.18 [2.14]	(1) 0.021 [0.018] (2) < 0.001 [< 0.001]
RBD (at baseline)	(1): > 7 (base: ≤7)	4.96	0.006	(1) > 7 (base: ≤7)	1.01 [0.94]	0.028 [0.039]
STAI (at baseline)	(1) 92 < STAI ≤ 102 (base: otherwise)	2.42	0.032	—	—	—

Numbers in square brackets are for RoCD > 0.02 month^−1^ without the GRS variable. Samples: 240 participants (RoCD > 0.11 month^−1^); 241 participants (RoCD > 0.02 month^−1^).

Each category of each variable in the logistic regression models (Table 3) was assigned a score that was equal to the regression coefficient for this category, rounded to the nearest half-integer or integer (Table 4). Any base categories were assumed to correspond to a zero score (Table 4). Patient’s PDCD score for prediction of RoCD > 0.11 month^−1^ (or RoCD > 0.02 month^−1^) was then defined as a sum of all respective category scores. Rounding of the category scores to the nearest integer was considered to be less accurate, but more convenient in the clinical practice, as only integer numbers are to be summed in this case to calculate the PDCD score(s). This is similar to how the Framingham Risk Score and DRAGON score are calculated^31–33^.

**Table 4:** Guideline for calculation of the PDCD scores for RoCD > 0.11 month^−1^ and RoCD > 0.02 month^−1^ for PD patients.

	RoCD > 0.11 month^−1^ Categories	(*PDCD*_*s*_)_0.5_	(*PDCD*_*s*_)_1_	RoCD > 0.02 month^-1^ Categories	(*PDCD*_*m*_)_0.5_	(*PDCD*_*m*_)_1_
**Predictor**						
Aβ_42_ (pg/ml)	(1) < 255 (base: ≥255)	2.5 0	3 0	(1) < 420 (base: ≥420)	1 0	1 0
α-syn (pg/ml)	(1) < 1060 (base: ≥1060)	8 0	8 0	(1) < 1950 (base: ≥ 1950)	1 0	1 0
t-tau (pg/ml)	(1) > 75 (base: ≤75)	5.5 0	6 0	(1) > 60 (base: ≤ 60)	1.5 0	1 0
p-tau (pg/ml)	(1) ≥ 17 (base: < 17)	4.5 0	5 0	—	—	—
p-tau/t-tau	(1) < 0.55 (base: ≥ 0.55)	7 0	7 0	(1) < 0.3 (base: ≥ 0.3)	0.5 0	1 0
UPDRS_1–3_ (at baseline)	(1) > 49 (base: ≤49)	4 0	4 0	(1) > 43 (base: ≤ 43)	1 0	1 0
GDS (at baseline)	(1) > 3 (base: ≤3)	3 0	3 0	(1) > 1 (base: ≤ 1)	0.5 0	1 0
Education (years)	(1) ≤ 10 (base: > 10)	4 0	4 0	(1) ≤ 12 (base: > 12)	1 0	1 0
GRS (at baseline)	—	—	—	(1) < – 0.01 (base: ≥ – 0.01)	1 [0] 0	1 [0] 0
MoCA_b_ (at baseline)	—	—	—	(1) > 27 (base: ≥ 27)	1 0	1 0
Age (years)	—	—	—	(1) 52 < Age ≤ 62 (2) Age > 62 (base: ≤ 52)	1.5 2 0	1 2 0
RBD (at baseline)	(1) > 7 (base: ≤7)	5 0	5 0	(1) > 7 (base: ≤ 7)	1 0	1 0
STAI (at baseline)	(1) 92 < STAI ≤ 102 (base: otherwise)	2.5 0	2 0	—	—	—
Maximum total		46	47		11.5 [10.5]	12^11^
Cut-off		17.5	18		5.5 [4.5]	6^5^

Cut-offs for the PDCD scores are shown in the last row. The indices 0.5 and 1 in the PDCD notations indicate rounding of the regression coefficients to the nearest half-integer and nearest integer, respectively. Numbers in square brackets are for the *PDCD*_*m*_ scores without the GRS variable. Samples: 240 participants (RoCD > 0.11 month^−1^); 241 participants (RoCD > 0.02 month^−1^).

Table 4 is a guideline for the clinical determination of the PDCD scores. For each patient, the clinical and pathological variables fall within one of the categories shown in Table 4. This determines the respective category scores for the patient. Sum of all these scores gives the respective PDCD score. For example, if for a patient Aβ_42_ = 300 pg/ml; α-syn = 1500 pg/ml; t-tau = 100 pg/ml; p-tau = 20 pg/ml; p-tau/t-tau = 0.2; UPDRS_1–3_ = 40; GDS = 2; Education = 25 years; RBD = 10; and STAI = 110, then the corresponding PDCD scores for severe rate of cognitive decline are calculated as (Table 4):

$${(PDC{D}_{s})}_{0.5}=0+0+5.5+4.5+7+0+0+0+5+0=22;$$

$${(PDC{D}_{s})}_{1}=0+0+6+5+7+0+0+0+5+0=23.$$

Using this procedure, the PDCD scores were calculated for all study participants. ROC regression analysis was then used to validate the PDCD scores as new integrated biomarkers, and to determine their sensitivities and specificities in predicting RoCD > 0.11 month^−1^ and RoCD > 0.02 month^−1^ (Table 2). It can be seen that the sensitivities and specificities for the RoCD scores are close to (or even better than) those for *M*_*s*_ and *M*_*m*_ (Table 2). This illustrates high level of stability of the obtained outcomes and the appropriateness of the adopted categorization procedure.

Rounding to the nearest integer might be expected to give less accurate outcomes compared to rounding to the nearest half-integer. At the same time, there are no differences between the values of AUC for the PDCD markers with rounding to the nearest half integer or integer (Table 2). This is a further illustration of stability of the developed PDCD scores as PD progression biomarkers on the considered sample of participants.

Note that sensitivities and specificities for the PDCD scores in Table 2 are presented for the cut-off values of the respective scores (Table 4). Some fluctuations of the sensitivities and specificities for the *PDCD*_*m*_ scores in Table 2 are caused by the discrete nature of the scores (Table 4) and relatively low values for the discrete cut-off scores. In this case, alterations in the cut-off values (e.g., due to rounding to the nearest half integer instead of the nearest integer, or due to discarding the GRS variable – Table 4) cause notable variations in the resultant sensitivities and specificities of the markers.

The sensitivities and specificities for the *PDCD*_*s*_ integrated markers are notably higher than those for the *PDCD*_*m*_ markers (Table 2). This is particularly important for accurate identification of PD patients with high risk of developing of severe cognitive decline, which will be important for more accurate clinical advice and targeted development of clinical trials. Further, it is also important to observe that the areas under the ROC curves for the *PDCD*_*s*_ and *PDCD*_*m*_ markers (Table 2) are about the same (for the *PDCD*_*m*_ markers), and significantly higher (for the *PDCD*_*s*_ markers), compared to those for the widely accepted Framingham Risk Score (below 0.75^32^) and DRAGON score (around 0.84^33^). This is a further indication of the practical usefulness of the developed PDCD scores for prediction of possible rates of cognitive decline in early-stage PD patients.

**Figure 4:**
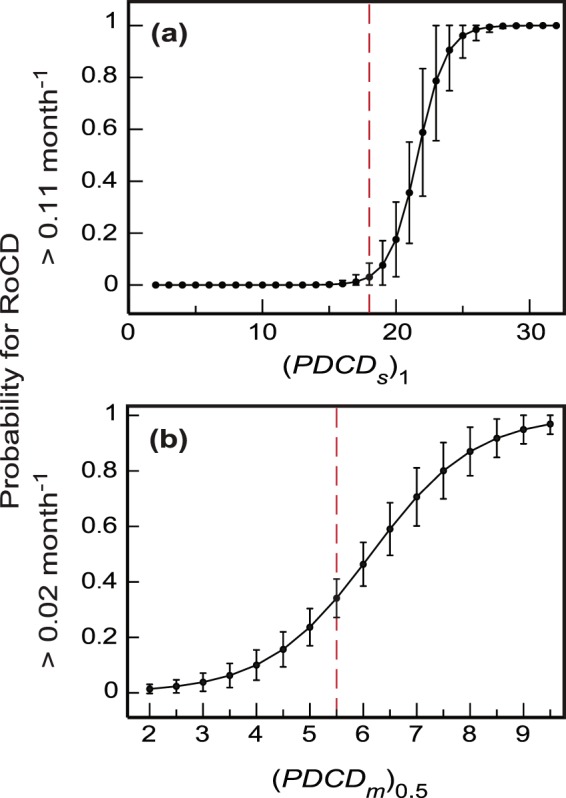
Probabilities for a PD patient to experience cognitive declines with: (**a**) RoCD > 0.11 month^−1^ (severe cognitive decline); and (**b**) RoCD > 0.02 month^−1^ as functions of the values of the respective PDCD scores. The vertical bars show the 95% prediction intervals for the corresponding predicted probabilities. For a similar probability graph for RoCD > 0.02 month^−1^ without the GRS variable see Supplementary Fig. 4.

The cut-off value for *PDCD*_*m*_ score (Table 4) separates PD patients for whom RoCD is likely to exceed 0.02 month^−1^ from those who are unlikely to experience such decline. Similarly, the cut-off values for *PDCD*_*s*_ scores (Table 4) separate patients who are likely to experience severe cognitive decline (RoCD > 0.11 month^−1^) from those who are unlikely to experience such decline. For an ideal marker (with the 100% sensitivity and specificity), the cut-off point perfectly separates the two groups of people that this marker is supposed to identify. If the sensitivity and specificity are below 100%, exceeding a PDCD score cut-off does not necessarily mean that the respective cognitive decline is inevitable. A better and more accurate understanding of risks for PD patients to experience cognitive decline can be obtained from the probability graphs (Fig. 4).

The probability graphs are an essential tool for simple clinical evaluation of risks for a PD patient to experience severe or mild-to-moderate cognitive decline. For example, if (*PDCD*_*m*_)_0.5_ = 8, then the probability for this patient to experience cognitive decline with RoCD > 0.02 month^−1^ is nearly 90% (Fig. 4b). In this case, (*PDCD*_*s*_)_1_ should be calculated to check if the patient is likely to experience severe cognitive decline with RoCD > 0.11 month^−1^. If the patient scores, for example, 20 points on the (*PDCD*_*s*_)_1_ scale, then the probability for him/her to experience severe cognitive decline is around 20% (Fig. 4a). However, if the patient’s score on the (*PDCD*_*s*_)_1_ scale is 17, then the probability that this patient will experience severe cognitive decline is rather negligible (Fig. 4a), and it should be concluded that he/she is likely to have mild-to-moderate decline with 0.02 month^−1^ < RoCD ≤ 0.11 month^−1^ (with the probability of nearly 90%, as indicated above).

The sequence of using the two developed PDCD scores could be reversed, and the (*PDCD*_*s*_)_1_ score could be used first to check if the patient is likely to have severe cognitive decline (RoCD > 0.11 month^−1^). If the probability of this is low, then the (*PDCD*_*m*_)_0.5_ score should be checked to see if the patient is likely to develop mild-to-moderate cognitive decline with 0.02 month^−1^ < RoCD ≤ 0.11 month^−1^.

## Conclusions

This study has conducted consistent consideration and analysis of 19 baseline clinical, pathological and demographic variables to develop and optimize the effective integrated biomarkers for prediction of progression of PD in the form of severe or mild-to-moderate cognitive declines. Severe cognitive decline was considered to occur where RoCD > 0.11 month^−1^, corresponding to more than 5 point decline on the MoCA scale within 4 years. Mild-to-moderate cognitive decline was considered to occur where 0.02 month^−1^ < RoCD ≤ 0.11 month^−1^, which is approximately between 1 and 5 points decline on the MoCA scale in 4 years.

Sensitivities and specificities of the integrated biomarkers exceeded 90% for prediction of severe cognitive decline, demonstrating the excellent capacity of these biomarkers to resolve the long-standing clinical issue with unreliable prognosis of cognitive decline in PD. This will aid with the development of effective targeted treatments and clinical trials.

Recognizing difficulties with use of complex computational tools in the clinical practice, the current study proposed, developed and validated (as new integrated biomarkers) the Parkinson’s disease cognitive decline scores. These scores are similar to the previously developed and widely recognized Framingham Risk Score for cardiovascular conditions^31,32^ and DRAGON score for prognosis of acute ischemic stroke patients^33^.

The PDCD scores demonstrated exceptional capabilities and stability in predicting the severe cognitive decline in PD, with the sensitivities and specificities around 93%. Combined with the simplicity of their determination in the clinical environment (provided that the measures constituting the markers are available), these scores are expected to be an invaluable tool for neurologists in their evaluation of PD progression and selection of optimal treatments and management approaches.

The main limitations of the study and obtained outcomes include the reliance on a single PPMI cohort of 269 participants. Future multi-cohort validation of the developed biomarkers and PDCD scores will be beneficial. The study was limited to the 22 variables and measures available from the PPMI database, although the developed approach is not limited to these particular variables. Nineteen of the 22 parameters were systematically considered, while the consideration of EGF, cholesterols and triglycerides was rather limited due to their sample size limitations. Future involvement of other relevant parameters and measures may be useful for further improvement of the integrated progression biomarkers and PDCD scores. All study participants with the severe cognitive decline were older than 60 years. This highlights a possible limitation of the considered sample with regard to the Age variable – it is unclear whether the age of 60 years is a threshold for the possibility of the severe cognitive decline or this is a sample limitation, which further highlights the need for further investigation of this question and consideration of other PD cohorts. The determination of the rate of cognitive decline was based on the MoCA scale for general cognition. This should be considered as another potential limitation, although the MoCA scale is widely recognized as a valid instrument for evaluation of the general cognition functions including in PD patients^13,21,29,30,39^. The period of observation of the study participants was for the duration between 4 years and 6 years after the initial screening visit. Taking into account that all participants were diagnosed with idiopathic PD within 2 years prior to the initial screening visit, the obtained predictions of the likelihood of progression of cognitive decline may not extend significantly beyond 6–8 years after PD diagnosis. Finally, the PPMI baseline data was collected within 2 years after the initial PD diagnosis. Therefore, this is the earliest stage at which the scoring system developed in the current paper was designed to predict possible cognitive decline among PD patients. Earlier prognosis would require the analysis of other databases collected at earlier stages (e.g., during prodromal stages of PD), which is beyond the scope of the current paper.

The developed approach to the determination of the PDCD scores has general neurology implications. It should be applicable to the development of similar clinical scores for PD diagnostics, progression of its motor symptoms, as well as for other neurodegenerative conditions with significant heterogeneity and prognostic/diagnostic difficulties including dementia with Lewy bodies, multiple system atrophy, Alzheimer’s disease, multiple sclerosis, etc.

## Methods

### Statistical methodology

The analysis was conducted using the Stata14 software package^40^. Logistic regressions were used to determine predictor variables with significant effects on the probabilities for participants to experience RoCD > 0.02 month^−1^ and RoCD > 0.11 month^−1^. As explained in the Section ‘Variables’ above and in the Supplementary Information (Supplementary Fig. 1), these category boundaries for RoCD were chosen to ensure reasonable numbers of participants in each category (around 10% with RoCD > 0.11 month^−1^ and around 30% with 0.02 month^−1^ < RoCD ≤ 0.11 month^−1^). In addition, the adopted RoCD categories also ensured high levels of statistical significance of the effects of the considered predictor variables in the developed logistic regression models. Other options for choosing the category boundaries for the RoCD variable are also considered in the Supplementary Information (Supplementary Fig. 2).

The large number of predictors having potential impacts on RoCD caused difficulties with understanding of which variables and/or their combinations should be involved in the integrated biomarkers. Therefore, model-averaging procedure was used to determine relative variable importance for each of the markers^41^. In this procedure, more than 500000 logistic regression models (e.g., for the probability to experience RoCD > 0.11 month^−1^) with all possible combinations of the clinical and pathological measures were computed, and the fit for each model evaluated using the Akaike Information Criterion^41^. An Akaike weight for a model is the probability that this model is the true (most probable) model. Relative importance of a variable is calculated as a sum of the Akaike weights for all models involving this variable^41^. It is equal to the probability that the variable appears in the most probable model. If this probability (relative variable importance) is large, then the variable is likely to be important for prediction of the considered cognitive decline. Sets of most important predictors were found for severe and mild-to-moderate cognitive declines in PD.

The sets of most important predictors were the starting point for the development of logistic regressions for prediction of risks of RoCD > 0.11 month^−1^ and RoCD > 0.02 month^−1^. Further variable categorizations were also considered and used (for some of the variables) to ensure better levels of statistical significance of any predictor variables in the developed multiple logistic regressions. Such categorization allowed taking into account possible non-linearities of the effects of some of the predictor variables. Linear combinations of the significant variables in the logistic regressions, weighed by their respective regression coefficients, were considered as the integrated biomarkers for prediction of cognitive decline in PD. These biomarkers were analyzed using the receiver operating characteristic (ROC) regression analysis^42^, including the respective cut-off values, sensitivities, specificities, and their prediction intervals.

In each of the developed models, only participants with full sets of the significant predictor variables were considered, and any participants with missing values of one or more significant variables involved in the model were automatically removed from the model sample. As a result, the actual samples for the developed models varied (as indicated above for each model), depending on the numbers of discarded participants with missing values of significant variables. The observation numbers for all considered predictor variables are shown in Supplementary Table 1. Cross-validations of the developed integrated biomarkers *M*_*s*_ and *M*_*m*_ were conducted using bootstrapping re-samplings for the ROC regressions^41,43^ - see the Supplementary Information for more detail.

### Variants for calculating genetic risk score (GRS)

GRS was calculated by summing the risk allele counts for the 30 variants associated with risk of PD, which are shown in Table 5, including the respective nearest genes^27,38^.

**Table 5:** The 30 variants (single nucleotide polymorphisms) associated with risk of PD, including the respective nearest genes^27,38^.

rs ID	Nearest gene(s)
rs114138760	GBA/SYT11
rs76763715	GBA
rs71628662	GBA/SYT11
rs823118	RAB7L1/NUCKS1
rs10797576	SIPA1L2
rs6430538	ACMSD/TMEM163
rs1955337	STK39
rs12637471	MCCC1
rs34884217	TMEM175/GAK/DGKQ
rs34311866	TMEM175/GAK/DGKQ
rs11724635	BST1
rs6812193	FAM47E/SCARB2
rs356181	SNCA
rs3910105	SNCA
rs8192591	HLA-DQB1
rs115462410	HLA-DQB1
rs199347	GPNMB
rs591323	FGF20
rs118117788	INPP5F
rs329648	MIR4697
rs76904798	LRRK2
rs34637584	LRRK2
rs11060180	CCDC62
rs11158026	GCH1
rs2414739	VPS13C
rs14235	BCKDK/STX1B
rs11868035	SREBF/RAI1
rs17649553	MAPT
rs12456492	RIT2
rs55785911	DDRGK1

### Ethical approval

The PPMI study and protocols were approved by the appropriate local ethics committees and review boards at the 24 enrolment sites (18 in the US, 5 in Europe, and 1 in Australia), including the provision of written informed consent to participate from all participants^35,44^. The methods in the current study were carried out in accordance with the ethical standards laid down in the 1964 Declaration of Helsinki and its later amendments. The approval for the use of the data in the current study was given by the Parkinson’s Progression Markers Initiative.

## Supplementary information

Parkinson’s disease prognostic scores for progression of cognitive decline

## Data Availability

The data used in the preparation of this article is available from the Parkinson’s Progression Markers Initiative (PPMI) database (www.ppmi-info.org/data).
