# Publisher Correction: Parkinson’s disease prognostic scores for progression of cognitive decline

**DOI:** 10.1038/s41598-020-76437-z

**Published:** 2021-03-09

**Authors:** Galina Gramotnev, Dmitri K. Gramotnev, Alexandra Gramotnev

**Affiliations:** 1 Research and Data Analysis Centre, GPO Box 1272, Aspley, QLD 4034 Australia; 2 University of Sunshine Coast, Thompson Institute, 12 Innovation Parkway, Birtinya, QLD 4575 Australia

Correction to: *Scientific Reports* 10.1038/s41598-019-54029-w, published online 25 November 2019

In the original PDF version of this Article, there were typesetting errors in Equations 1 and 2 where dash signs were incorrectly typeset as minus signs. As a result, the symbols ‘t-tau’, ‘p-tau’, ‘α-syn’ and ‘1-3’ were incorrectly typeset as ‘t – tau’, ‘p – tau’, ‘α – syn’ and ‘1 – 3’ respectively. Secondly, in Equation 2 the notation for amyloid was incorrectly typeset as Ab_42_. The incorrect versions of Equations 1 and 2 appear below:

Equation (1)

$$ \begin{aligned} M_{m} = & -\, 0.00559 \times A\beta_{42} + 1.05\left( {{\text {if}}\,\upalpha - {\text {syn}} < 1950 \,\text {pg/ml}} \right) \\ &\, +\, 1.17\left( {\text {if}\, \text t - \text {tau} > 60\, \text {pg/ml}} \right) - 8.89 \times \frac{\text p - \text {tau}}{{\text t - \text {tau}}} + 7.63 \times \left( {\frac{\text p - \text {tau}}{{\text t - \text {tau}}}} \right)^{2} \\ &\, +\, 0.257 \times \text {MoCA}_{\text b} + 0.0302 \times \text {UPDRS}_{1 - 3} + 0.161 \times \text {RBD} \\ & \,+ \,0.132 \times \text {GDS} - 0.151 \times \text {Education} - 13.53937 \times \text {Age} \\ &\, +\, 0.3564239 \times \text {Age}^{2} - 0.0040239 \times \text {Age}^{3} + 0.0000166 \times \text {Age}^{4} \\ &\, +\, 0.758 \times \text {GRS} \\ \end{aligned} $$

Equation (2)

$$ \begin{aligned} M_{s} = & -\, 0.0156 \times \text {Ab}_{42} - 0.00363 \times \upalpha - \text {syn} + 0.150 \times \text t - \text {tau} \\ & \,+\, 2.66\left( {\text {if p} - \text{tau} \ge 17\, \text {pg/ml}} \right) + 0.0633 \times \text {UPDRS}_{1 - 3} + 0.339   \times\, \text {RBD} \\ & +\, 0.457 \times \text {GDS} + 0.109 \times \text {STAI} - 3.64\left( {\text {if female}} \right) \\ & +\, 2.94 \times \text {DaT}_{\text p} . \\ \end{aligned} $$

Furthermore, in the original HTML and PDF versions of this Article there were errors in the (*PDCD*_*m*_)_1_ column of Table 4. In the row “Maximum total”,

“12^11^”

now reads:

“12 [11]”

In the row “Cut-off”,

“6^5^”

now reads:

“6 [5]”

These errors have now been corrected in the PDF and HTML versions of the Article.

