# Supplementary material for: Parkinson’s disease prognostic scores for progression of cognitive decline
Source: Sci Rep. 2019 Nov 25;9:17485. doi: 10.1038/s41598-019-54029-w (PMC6877592; doi:10.1038/s41598-019-54029-w)
Supplement: Supplementary file 1 — Parkinson’s disease prognostic scores for progression of cognitive decline [file 41598_2019_54029_MOESM1_ESM.pdf]

# Parkinson's disease prognostic scores for progression of cognitive decline

Galina Gramotnev, Dmitri K. Gramotnev, Alexandra Gramotnev

## Supplementary Information

### Categorization of rate of cognitive decline

Supplementary Figure 1 shows the percentages of participants in the considered sample, for whom RoCD values exceeded the values of the rate of cognitive decline $RoCD_0$ shown on the horizontal axis. For ease of reference, the same figure also shows the 4-year declines in the MoCA score (right vertical axis) corresponding to the indicated values of $RoCD_0$. It can be seen that just under 10% of patients with early stages of PD had RoCD > 0.11 month$^{-1}$ (Supplementary Figure 1), which corresponds to the average decline of their MoCA scores by more than 5 points in 4 years. Such cognitive decline was regarded as severe, and it is likely to cause cognitive impairment within around 4 years, even where the baseline MoCA score ($MoCA_b$) is high. At the same time, the values of RoCD between 0.02 month$^{-1}$ and 0.11 month$^{-1}$ (corresponding to the 4-year decline in the MoCA score between 1 and 5 points) were regarded as mild-to-moderate cognitive decline. The number of participants with RoCD > 0.02 month$^{-1}$ was about 38% (Supplementary Figure 1). This illustrates reasonable ground for the adopted categorization of the RoCD variable into the severe rate of cognitive decline (with RoCD > 0.11 month$^{-1}$, occurring in ~ 10% of newly diagnosed patients with PD) and mild-to-moderate cognitive decline (with 0.02 month$^{-1}$ < RoCD ≤ 0.11 month$^{-1}$, occurring in ~ 30% of newly diagnosed patients with PD).

The integrated biomarkers $M_m$ and $M_s$ (Eqs. (1) and (2)) were then developed to determine the risks of mild-to-moderate and severe cognitive declines among newly diagnosed PD patients. Nonetheless, a question remains about how would the

optimized sensitivities and specificities for optimally constructed integrated biomarkers change if the boundary values $ROCD_0$ for the adopted RoCD categories are different from 0.02 $month^{-1}$ and 0.11 $month^{-1}$? To answer this question, we further considered two other values of $RoCD_0$ = 0.06 $month^{-1}$ and 0.09 $month^{-1}$ and determined the sensitivities and specificities of the integrated biomarkers determining the risks of RoCD > 0.06 $month^{-1}$ and RoCD > 0.09 $month^{-1}$ – Supplementary Figure 2. For each of these boundary values of $RoCD_0$, optimized combinations of significant variables and measures were determined and used.

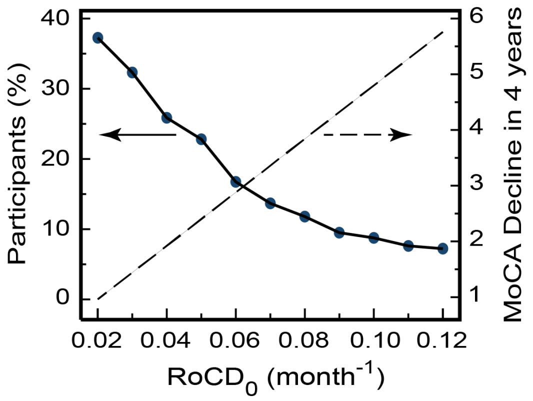


**Supplementary Figure 1.** Percentage of participants (left vertical axis) with RoCD > $RoCD_0$ shown on the horizontal axis. Dashed line shows the relationship between $RoCD_0$ and the corresponding 4-year decline of the MoCA score (right vertical axis).

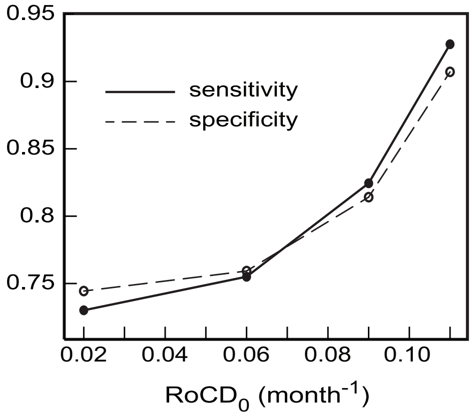


**Supplementary Figure 2.** The dependences of the sensitivities (solid curve) and specificities (dashed curve) on $RoCD_0$ values for the optimally constructed integrated biomarkers determining the risks of RoCD > $RoCD_0$.

It can be seen that the sensitivities and specificities of the optimized integrated biomarkers monotonically (albeit non-linearly) increase from their values for $M_m$ for $RoCD_0 = 0.02$ month$^{-1}$ to the values for $M_s$ for $RoCD_0 = 0.11$ month$^{-1}$ (Supplementary Figure 2). This further corroborates the consistency of the obtained outcomes and provides information about the dependence of the biomarker characteristics on assumed boundary values for the adopted RoCD categories.

**Summary statistics for predictor variables**

The current study considered 22 baseline predictor variables available from the PPMI database for 269 untreated participants with recent diagnosis of PD (given within 2 years prior to the baseline).

| Variable (baseline) | | Observations | Mean | Median | St. Dev. |
|---|---|---|---|---|---|
| $MoCA_b$ | | 269 | 27.09 | 28 | 2.31 |
| $A\beta_{42}$ (pg/ml) | | 261 | 366.00 | 363.4 | 101.08 |
| α-syn (pg/ml) | | 261 | 1840.18 | 1709.13 | 796.06 |
| t-tau (pg/ml) | | 258 | 44.02 | 40.55 | 17.75 |
| p-tau (pg/ml) | | 259 | 15.69 | 11.9 | 10.97 |
| p-tau / t-tau | | 256 | 0.3701 | 0.2962 | 0.2278 |
| IGF-1 (ng/ml) | | 264 | 136.98 | 124.8 | 54.50 |
| Prior Education (years) | | 269 | 15.62 | 16 | 2.89 |
| $UPDRS_{1\text{–}3}$ | | 267 | 31.85 | 30 | 13.37 |
| Age (years) | | 269 | 61.89 | 62.58 | 9.76 |
| RBD | | 269 | 4.14 | 4 | 2.63 |
| UPSIT | | 268 | 21.41 | 22 | 8.05 |
| GDS | | 267 | 2.30 | 2 | 2.38 |
| $DaT_p$ (putamen average) | | 259 | 0.8058 | 0.78 | 0.2717 |
| $DaT_c$ (caudate average) | | 259 | 1.9687 | 1.935 | 0.5294 |
| STAI | | 267 | 93.00 | 94 | 8.04 |
| GRS | | 250 | – 0.0161 | – 0.0165 | 0.0088 |
| EGF (pg/ml) | | 123 | 44.16 | 33.72 | 38.92 |
| Cholesterols (mg/dl) | | 123 | 188.61 | 187 | 41.53 |
| Triglycerides (mg/dl) | | 123 | 113.09 | 101.67 | 34.82 |
| Gender | Male (base) | 183 | - | | - |
| | Female | 86 | - | | - |
| DA | Never (base) | 179 | - | | - |
| | Yes | 90 | - | | - |

**Supplementary Table 1.** Summary statistics describing the 22 baseline variables for the considered sample of 269 recently diagnosed untreated PD patients.

Ten participants in the considered sample did not have their baseline DaT measures, and their DaT scans (showing dopamine deficiency) were obtained within the subsequent couple of months. For consistency in the current study, these 10 participants were regarded as missing their baseline DaT variables (Supplementary Table 1), but they were otherwise included in the considered sample, as they did have dopamine deficiency evidenced through DaT scans shortly after the baseline.

**Cross-validation**

Cross-validations of the developed integrated biomarkers $M_s$ and $M_m$ (Eqs. (1) and (2)) were conducted using 1000 bootstrapping re-samplings for the ROC regressions [41,44] for each of the two biomarkers – the outcomes shown in Supplementary Figures 3a and 3b, respectively. The cut-off points shown in Supplementary Figures 3a,b correspond to the following values: $M_{sc}$ = 15.34 (Se = 93%; Sp = 92%) and $M_{mc}$ = – 179.67 (Se = 74%; Sp = 77%). Comparing these values of the biomarker cut-offs and the corresponding sensitivities and specificities obtained from the bootstrapping approach with those presented in Fig. 3 and Table 2 confirms validity of the obtained outcomes for the integrated biomarkers $M_s$ and $M_m$.

Further, the values of AUC for the ROC regression curves obtained using the 1000 bootstrapping re-samplings (Supplementary Figures 3a,b) are 0.973 and 0.812, respectively. These values are also in excellent agreement with those given in Table 2, which is another illustration of validity of the integrated biomarkers $M_s$ and $M_m$.

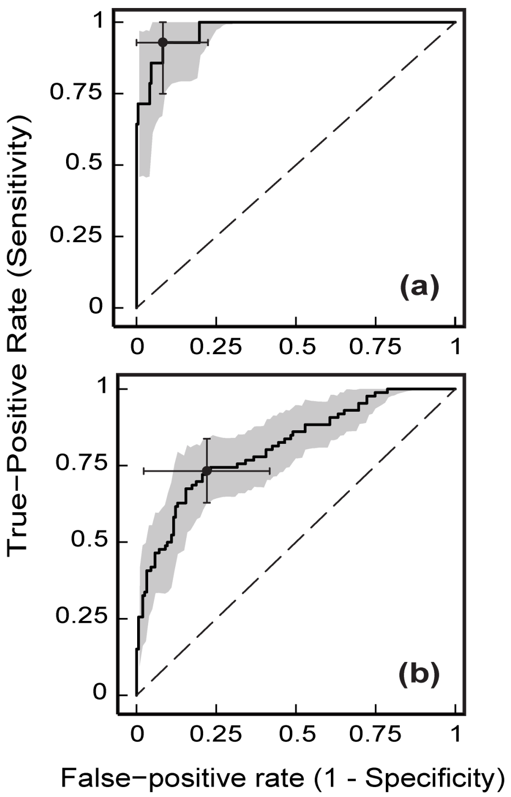


**Supplementary Figure 3.** The outcomes of the bootstrapping cross-validation of the integrated biomarkers $M_s$ and $M_m$ applied to the ROC regressions with the specificity step of 0.01 for the two considered characteristic rates of cognitive decline: (a) RoCD > 0.11 $month^{-1}$ (the $M_s$ biomarker), and (b) RoCD > 0.02 $month^{-1}$ (the $M_m$ biomarker). The shaded bands show the respective 95% confidence intervals for the sensitivities. The vertical error bars show the 95% confidence intervals for the sensitivities at the cut-off points, and the horizontal error bars show the 95% confidence intervals for the specificities at the cut-off points.

**PDCD probability graph in the absence of GRS**

As discussed in the main body of the paper, the GRS variable may not always be available to clinical practitioners and neurologists. Therefore, the probability dependence in Supplementary Figure 4 determines the risk for a person recently diagnosed with PD to experience RoCD > 0.02 $month^{-1}$ as a function of this person's $(PDCD_m)_{0.5}$ in the absence of the GRS variable.

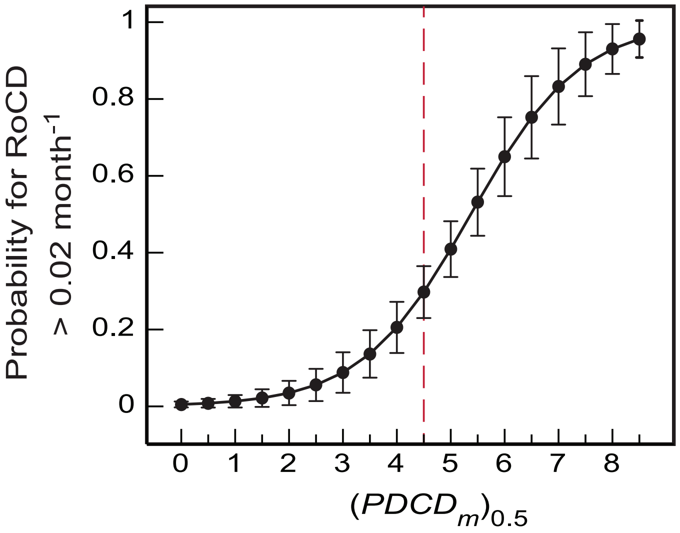


**Supplementary Figure 4.** The dependence of the probabilities for a PD patient to experience cognitive decline with RoCD > 0.02 month$^{-1}$ as a function of this patient's $(PDCD_m)_{0.5}$ in the absence of the GRS variable. The vertical bars show the 95% prediction intervals for the corresponding predicted probabilities.
